# Utility of ^18^ F-FDG and ^11^C-PBR28 microPET for the assessment of rat aortic aneurysm inflammation

**DOI:** 10.1186/s13550-014-0020-z

**Published:** 2014-05-10

**Authors:** Sean J English, Jose A Diaz, Xia Shao, David Gordon, Melissa Bevard, Gang Su, Peter K Henke, Virginia E Rogers, Gilbert R Upchurch, Morand Piert

**Affiliations:** Conrad Jobst Vascular Research Laboratories, University of Michigan Health System, Ann Arbor, MI 48109 USA; Division of Nuclear Medicine, Department of Radiology, University of Michigan Health System, Ann Arbor, MI 48109 USA; Department of Pathology, University of Michigan Health System, Ann Arbor, MI 48109 USA; Division of Vascular and Endovascular Surgery, University of Virginia Health System, Charlottesville, VA 22903 USA

**Keywords:** ^18^ F-FDG, ^11^C-PBR28, Positron emission tomography, Inflammation, Abdominal aortic aneurysm, Macrophage

## Abstract

**Background:**

The utility of ^18^ F-FDG and ^11^C-PBR28 to identify aortic wall inflammation associated with abdominal aortic aneurysm (AAA) development was assessed.

**Methods:**

Utilizing the porcine pancreatic elastase (PPE) perfusion model, abdominal aortas of male Sprague-Dawley rats were infused with active PPE (APPE, AAA; N = 24) or heat-inactivated PPE (IPPE, controls; N = 16). Aortic diameter increases were monitored by ultrasound (US). Three, 7, and 14 days after induction, APPE and IPPE rats were imaged using ^18^ F-FDG microPET (approximately 37 MBq IV) and compared with ^18^ F-FDG autoradiography (approximately 185 MBq IV) performed at day 14. A subset of APPE (N = 5) and IPPE (N = 6) animals were imaged with both ^11^C-PBR28 (approximately 19 MBq IV) and subsequent ^18^ F-FDG (approximately 37 MBq IV) microPET on the same day 14 days post PPE exposure. In addition, autoradiography of the retroperitoneal torso was performed after ^11^C-PBR28 (approximately 1,480 MBq IV) or ^18^ F-FDG (approximately 185 MBq IV) administration at 14 days post PPE exposure. Aortic wall-to-muscle ratios (AMRs) were determined for microPET and autoradiography. CD68 and translocator protein (TSPO) immunohistochemistry (IHC), as well as TSPO gene expression assays, were performed for validation.

**Results:**

Mean 3 (p = 0.009), 7 (p < 0.0001) and 14 (p < 0.0001) days aortic diameter increases were significantly greater for APPE AAAs compared to IPPE controls. No significant differences in ^18^ F-FDG AMR were determined at days 3 and 7 post PPE exposure; however, at day 14, the mean ^18^ F-FDG AMR was significantly elevated in APPE AAAs compared to IPPE controls on both microPET (p = 0.0002) and autoradiography (p = 0.02). Similarly, mean ^11^C-PBR28 AMR was significantly increased at day 14 in APPE AAAs compared to IPPE controls on both microPET (p = 0.04) and autoradiography (p = 0.02). For APPE AAAs, inhomogeneously increased ^18^ F-FDG and ^11^C-PBR28 uptake was noted preferentially at the anterolateral aspect of the AAA. Compared to controls, APPE AAAs demonstrated significantly increased macrophage cell counts by CD68 IHC (p = 0.001) as well as increased TSPO staining (p = 0.004). Mean TSPO gene expression for APPE AAAs was also significantly elevated compared to IPPE controls (p = 0.0002).

**Conclusion:**

Rat AAA wall inflammation can be visualized using ^18^ F-FDG and ^11^C-PBR28 microPET revealing regional differences of radiotracer uptake on microPET and autoradiography. These results support further investigation of ^18^ F-FDG and ^11^C-PBR28 in the noninvasive assessment of human AAA development.

**Electronic supplementary material:**

The online version of this article (doi:10.1186/s13550-014-0020-z) contains supplementary material, which is available to authorized users.

## Background

Abdominal aortic aneurysm (AAA) is a significant medical problem, with a high mortality rate, accounting for approximately 150,000 hospital admissions per year. AAA is the 10th leading cause of death in Caucasian men, ages 65 to 74 years and accounted for nearly 16,000 deaths overall. This equates to approximately 10% of males and approximately 2% of females, over the age of 60 years with a known AAA diagnosis. More than 50% of patients will present with a ruptured AAA (rAAA), without a previous diagnosis of AAA. Over 36,000 AAA repairs are performed a year in the USA, highlighting the financial impact of this disease. While clinically significant decreases in mortality following elective AAA repair have been documented, the mortality following open rAAA repair has remained high (50% to 75%) [1].

The primary factor considered for risk of human rAAA is the aortic diameter [2]. However, it is well documented that small AAAs (<5 cm) rupture, while many large AAAs (>8 cm) are incidentally discovered. Thus, there is no absolute diameter threshold at which AAAs rupture. This suggests that a new diagnostic modality needs to be developed to reliably identify an impending rupture. It is hypothesized that the final event leading to aortic rupture involves the degradation of collagen within the media, extending out through the adventitia. This occurs through a catalytic process induced by inflammatory cells in the aortic wall. Macrophages are a major source of key enzymes at play, particularly serine proteases, and are believed to play a critical role in this tissue degradation [3].

Inflammation of the vascular wall related to phagocytic macrophage activity can be demonstrated by increased uptake of 2-deoxy-2-[^18^ F]fluoro-d-glucose (^18^ F-FDG) PET in the arterial wall [4]. Accordingly, ^18^ F-FDG PET has been used to assess AAA wall inflammation [5], and increased ^18^ F-FDG uptake has been identified in symptomatic AAAs (pain on palpation of AAA) and with rapid aortic enlargement [6]. Recently, we were able to show that increased ^18^ F-FDG uptake is indeed related to an increased risk for rupture in the same established animal model utilized for this study [7].

Translocator protein (TSPO), formerly referred to as peripheral benzodiazepine receptor, is an 18-kDa outer mitochondrial protein highly expressed in phagocytic inflammatory cells, such as macrophages in the periphery and macrophage-like microglial cells in the central nervous system. It has been demonstrated that other inflammatory cells including neutrophils, B cells, natural killer, as well as CD4- and CD8-positive cells express TSPO to varying degrees [8]. The TSPO radioligand ^11^C-PBR28 ([methyl-^11^C]N-acetyl-N-(2-methoxybenzyl)-2-phenoxy-5-pyridinamine) has been used for neuroimaging with primates [9] and humans [10]. Other TSPO radioligands, including ^11^C-PK1195, have been utilized to evaluate macrophage presence and behavior in rodent and human carotid plaques [11,12].

In this study, ^18^ F-FDG and/or ^11^C-PBR28 microPET were evaluated to determine whether increased radiotracer uptake could identify aortic wall inflammation in rat AAAs. We utilized an established model, in which AAA development is induced by active porcine pancreatic elastase (APPE) infusion compared to heat-inactivated PPE (IPPE) controls [7]. PET imaging results were further supported and validated by ^18^ F-FDG and ^11^C-PBR28 autoradiography, as well as CD68 and TSPO immunohistochemistry (IHC) and cell culture experiments.

## Methods

Male Sprague-Dawley rats (200 to 300 g) were obtained from Charles River Laboratories (Wilmington, MA, USA) and utilized for all experiments. Animal anesthesia was performed with a mixture of approximately 1.5% isoflurane and oxygen for all procedures, respectively. The core body temperature was maintained with a heating pad (37°C). All procedures as listed in Table 1 were approved by the University of Michigan Universal Committee on the Use and Care of Animals (protocol number 10430).

**Table 1:** Study scheme: overview of the number of animals exposed to APPE or IPPE undergoing imaging

**Intervention**	**Day 0**	**Day 3**	**Day 7**	**Day 14**
PPE exposure	APPE *N* = 24			
	IPPE *N* = 16			
Ultrasound		APPE *N* = 8	APPE *N* = 15	APPE *N* = 24
		IPPE *N* = 4	IPPE *N* = 11	IPPE *N* = 16
^18^ F-FDG PET		APPE *N* = 7	APPE *N* = 7	APPE *N* = 9
		IPPE *N* = 10	IPPE *N* = 9	IPPE *N* = 6
^18^ F-FDG + ^11^C-PBR28 PET				APPE *N* = 5
				IPPE *N* = 6
^11^C-PBR28 autoradiography				APPE *N* = 4
				IPPE *N* = 4
				APPE *N* = 4
				IPPE *N* = 5

### AAA model

AAAs (N = 24) were established utilizing active porcine pancreatic elastase (APPE, 12 U/mL), while control animals (N = 16) were exposed to heat-inactivated porcine pancreatic elastase (IPPE 12 U/mL, APPE heated at 90°C for 45 min) as previously described [13]. After ventral abdominal wall incision, a customized polyethylene catheter (Braintree Scientific, Braintree, MA, USA) was introduced through an aortotomy, and PPE (12 U/mL) was instilled into the isolated aortic segment for 30 min. The exposed segment was dilated to a maximal diameter, and constant pressure was maintained with the use of a syringe pump. Using a video micrometer configured with NIS Elements software (Nikon, Melville, NY, USA), the aortic diameter was measured just distal to the crossing left renal vein and the proximal ligature, just proximal to the iliac bifurcation and the distal ligature, and midway between these two locations. During PPE exposure and aortic dilation, average increases in aortic diameter of 51.3 ± 2.3% and 48.2 ± 3.7% (p = not significant, NS) were observed for the APPE and IPPE groups, respectively. Upon reestablishment of segmental blood flow, mean aortic diameter increases of 47.0 ± 2.5% and 34.0 ± 3.1% (p = 0.003) were observed for the APPE and IPPE groups, respectively.

At the time of sacrifice, animals were anesthetized with isoflurane, and the ventral incision was reopened. The abdominal aorta was dissected free from the surrounding tissues. Blood was collected from the inferior vena cava, and the aorta was then excised from the level of the left renal vein to the iliac bifurcation and processed.

### Noninvasive aortic diameter measurements

Prior to performing a laparotomy, the intraluminal diameter of the aorta just distal to the left renal vein crossing, just proximal to the iliac bifurcation, and midway between these two locations was measured with ultrasound (US, 12 MHz Zonare, Mountain View, CA, USA). Percentage increases in aortic diameter were determined considering the average baseline intraluminal aortic diameter and the maximum aortic diameter at days 3, 7, and 14 post PPE exposure. An aortic aneurysm was defined by a >100% increase in the aortic diameter compared to pretreatment measurements.

### ^18^ F-FDG and ^11^C-PBR28 microPET

^11^C-PBR28 was synthesized as described in the literature [14,15]. At days 3, 7, and 14 after PPE exposure, sets of APPE and IPPE controls were imaged dynamically on a microPET R4 scanner (Concorde/Siemens Microsystems, Knoxville, TN, USA) for 90 min after IV injection of approximately 37 MBq ^18^ F-FDG [16]. To avoid interference from radiotracer pooling in the inferior vena cava, ^18^ F-FDG was injected intravenously via a right external jugular vein (EJV) cannula.

Separate sets of IPPE control (N = 6) and APPE AAA (N = 5) animals underwent imaging with both radiotracers. First, approximately 19 MBq of ^11^C-PBR28 was injected via the right EJV catheter, and a 60-min dynamic microPET study was started. While keeping the animal under anesthesia and after near complete decay of ^11^C-radioactivity (at 90 min post injection (p.i.) of ^11^C-PBR28), 37 MBq of ^18^ F-FDG was injected, and a 90-min dynamic scan was started.

### MicroPET image data reconstruction and analysis

All image data were corrected for attenuation by measured transmission scan, scatter, random events, and decay. Data were reconstructed using iterative ordered subset expectation maximization - maximum a posteriori (OSEM-MAP) [17] yielding a reconstructed image resolution of approximately 1.4 mm. To define volumes of interest (VOIs), first the abdominal aorta was identified on early bolus images (first 90 s), while summed transaxial late phase ^11^C-PBR28 (10 to 60 min p.i.) and late phase ^18^ F-FDG (60 to 90 min p.i.) uptake data were used for further analyses. The total AAA or control aortic radiotracer uptake was normalized to the mean adjacent psoas muscle uptake (AMR_PET_) for both ^11^C-PBR28 and ^18^ F-FDG data sets using ASI Pro VM software (Siemens Medical Systems, Malvern, PA, USA) [18]. Normalization to muscle was chosen as the normal thoracic aorta was generally not assessable due to the limited field of view during dynamic microPET imaging and because the adjacent psoas muscle was available for normalization on both microPET and autoradiography.

In addition, maximum intensity projection (MIP) images of the abdominal aorta with iliac bifurcation were created by zeroing all voxels outside of the aorta VOI, then rotating the image volume about the Z-axis in increments of 11.25° (360°/32°). At each rotation, the VOI pixels were weighted by a scale factor ranging linearly from 1.0 for the front-most voxel to 0.5 for the furthest-most voxel, and then the maximum intensity voxel was chosen for each pixel of the projection. Software to generate the MIP images was written using the IDL programming language (Exelis Visual Information Solutions, Boulder, CO, USA). Individual projections were volume rendered using Adobe After Effects CS5 software.

### ^11^C-PBR28 and ^18^ F-FDG autoradiography

For autoradiography, ^18^ F-FDG (approximately 185 MBq) was injected IV in five IPPE control and four APPE animals. ^11^C-PBR28 (approximately 1,480 MBq) was injected IV in five APPE and four IPPE controls. Circulation time prior to harvest was 60 min for ^18^ F-FDG and 10 min for ^11^C-PBR28, respectively. Given rapid ^11^C-PBR kinetics [15], this earlier time point was chosen to optimize count statistics (20.3 min physical half-life of ^11^C) bearing in mind that whole-mount sectioning for autoradiography typically required considerable time (5 to 7 half-lives of ^11^C) prior to exposure of the phosphor imaging screen. The infrarenal abdominal aorta with aortic bifurcation and surrounding musculature and vertebrae were harvested en bloc and quickly frozen in OCT (Sakura, Torrance, CA, USA) at −80°C for approximately 20 min. A Leica cryomicrotome (Leica Microsystems Inc, Buffalo Grove, IL, USA) was used to obtain 20-μm sections for whole-mount autoradiography. Block face photographs were taken during sectioning to correlate radiotracer uptake on autoradiography with anatomical landmarks. Radioactivity was determined using a calibrated bio-image analyzer BAS-1800 (FUJIFILM Life Science, Stamford, CT, USA) after exposing sections for 2 and 4 h in the case of ^11^C-PBR28 and ^18^ F-FDG, respectively. The number of photo-stimulated luminescence events per square millimeter (PSL/mm^2^, mean ± SD) corrected for background was measured using vendor-specific software (BAS-Reader) by drawing individual regions of interest (ROIs) for the aortic wall and psoas muscle, facilitated by comparison with block face photos for each section. Specifically, three autoradiographic coronal sections at the level of maximum aortic diameter were considered to calculate the mean autoradiographic aortic wall-to-psoas muscle uptake ratio (AMR_ARG_) for control and AAA animals for further analysis.

### Histology and immunohistochemistry

Tissues from the maximum diameter of each AAA and from a comparable region of each control aorta were taken for histologic analysis. Since the remaining tissue was utilized for molecular analyses, a direct spatial comparison between autoradiography and immunohistochemistry was not possible. Aortic tissue was fixed in 10% formaldehyde for 8 h, transferred and stored in 70% ethanol, and embedded in paraffin for sectioning (5 μm). Macrophage staining was performed with a mouse anti-rat CD68 primary antibody (1:67, Serotec, Raleigh, NC, USA), and visualization of anti-CD68 was done with DAB (Dako North America, Inc., Carpinteria, CA, USA), and counter staining was performed with hematoxylin 1 (Richard-Allan Scientific Co, Kalamazoo, MI, USA). TSPO IHC was performed with mouse anti-rat TSPO primary antibody (1:100, Santa Cruz Biotechnology, Inc, Santa Cruz, CA, USA). After using an antigen retrieval solution (Vector Laboratories, Burlingame, CA, USA), the primary antibodies were detected using a Vectastain Elite Kit (Vector Laboratories, Burlingame, CA, USA). Utilizing a Leica DMR microscope (Leica Microsystems IN, Buffalo Grove, IL, USA), motorized stage, Stereo Investigator software (version 4.34, Microbrightfield Inc., Williston, VT, USA), CD68- and TSPO-positive cell counts per high-power field (HPF) were determined (approximately 150 HPFs at × 63 magnification).

### Macrophage cell culture experiments

^18^ F-FDG and ^11^C-PBR28 uptake was determined in the mouse peritoneal RAW 264.7 macrophage cell line (ATCC, Manassas, VA, USA) maintained in complete RPMI 1640 (C-RPMI), and 100 units/mL penicillin/streptomycin. The cells were grown to approximately 80% confluence on polystyrene 6-well plates in C-RPMI. Sample (1.14 to 1.99 × 10^5^) cells were incubated for 10 min with approximately 90 kBq of ^11^C-PBR28 and approximately 390 kBq of ^18^ F-FDG (both at approximately 10 nmol/mL concentration), respectively. ^11^C-PBR28 and ^18^ F-FDG cell uptake values were determined in macrophages stimulated at 10, 100, and 1,000 μg/mL lipopolysaccharide (LPS, Sigma-Aldrich Corporation, St. Louis, MO, USA) for 24 h and compared to nonstimulated cultures. The cells were washed three times in ice-cold PBS using a semi-automatic cell harvester (model 48LT; Brandel, Gaithersburg, MD, USA) before counting. All experiments were performed in triplicate.

### Quantitative real-time polymerase chain reaction

Established techniques using TRIzol reagent (Invitrogen, Carlsbad, CA, USA) were used to extract TSPO mRNA for quantitative real-time polymerase chain reaction (qRT-PCR). Briefly, harvested aortic tissue was added to 1.0 mL TRIzol reagent, and the tissue was homogenized. Chloroform (99%, 0.2 mL) was added to the homogenized tissue, vortexed, and centrifuged, and the supernatant was transferred to Eppendorf tubes. RNA precipitation was performed with isopropanol (99%). The remaining mRNA pellet was washed with 75% ethanol and centrifuged. The supernatant was then aspirated off, and the pellet was dried at room temperature. The pellet was redissolved in nuclease-free water (Ambion®; Life Technologies, Grand Island, NY, USA) at 55°C. The RNA concentrations were then measured with a Nanodrop 2000 spectrophotometer (ThermoScientific, Pittsburgh, PA, USA). To produce 0.5 μg of cDNA, 0.5 μg/5 μL RNA was utilized for reverse transcription, with 1 μL iScript™ reverse transcriptase (iScript™ cDNA Synthesis Kit, Cat# 170-8891; Bio-Rad Corp. Hercules, CA, USA), 4 μL 5x iScript™ reaction mix (iScript™ cDNA Synthesis Kit, Cat# 170-8891, Bio-Rad) and 10 μL nuclease-free water (Cat# AM9937 Ambion®) in a thermocycler for DNA amplification device (Mastercycler® gradient 5331, Eppendorf, Westburg, NY, USA).

qRT-PCR was performed utilizing 10 ng/well of cDNA. Primers and the SYBR Green Master Mix used for qRT-PCR were obtained from SABiosciences (Qiagen, Frederick, MD, USA). mRNA expression of TSPO (catalog no. Rn00560890) was compared with that of 18S, a housekeeping gene (catalog no. Mm03928990). The CFX96 Real-Time System and the CXF Manager Software (version 2.1) was used to amplify target DNA and obtain the take-off values and melt curves. The following program was used on the Rotor Gene: 95°C for 10 min; 40 cycles of 95°C for 15 s and 60°C for 60 s.

### Data analysis

Statistical analyses were performed using Prism 5 (GraphPad Software, Inc., La Jolla, CA, USA). Quantitative results were analyzed by unpaired two-tailed unequal variance t tests (also known as Welch's correction) to account for possible unequal group variances [19]. Paired data were assessed using the Wilcoxon signed-rank test. Data are presented as mean ± standard error of mean (SEM), when appropriate, and p values of less than 0.05 were considered statistically significant.

## Results and discussion

### Results

#### Aortic diameters

**Figure 1:**
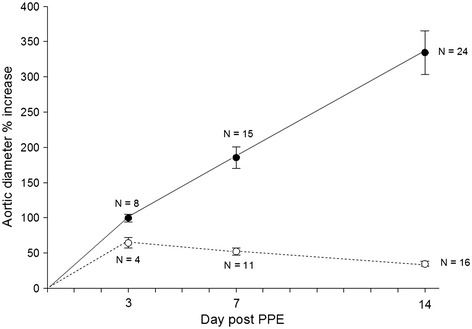
**Aortic diameter percent increases.** Active porcine pancreatic elastase (APPE) exposed animals (black circles and solid lines) developed abdominal aortic aneurysms (AAA) by day 3 post APPE exposure, and AAA diameters continued to increase over the 14-day time period. Heat-inactivated PPE (IPPE) exposed animals (white circles, dashed lines) did not develop AAAs (defined as a >100% increase in aortic diameter compared to pretreatment measurements).

The mean day 3 aortic diameter increases compared to pretreatment measurements for the APPE (N = 8) and the IPPE (N = 4) groups were 99.7 ± 5.5% and 64.4 ± 7.5% (p = 0.009), respectively. At days 7 and 14, the aortic diameter further increased significantly in the APPE group to 185.6 ± 15.2% (N = 15, p < 0.0001) and 335.0 ± 30.8% (N = 24, p < 0.0001), respectively. For the IPPE control group, the aortic diameter slightly decreased to 52.3 ± 5.0% (N = 11) and 34.6 ± 3.6% (N = 16) at days 7 and 14, respectively (Figure 1).

#### ^11^C-PBR28 and ^18^ F-FDG microPET

**Figure 2:**
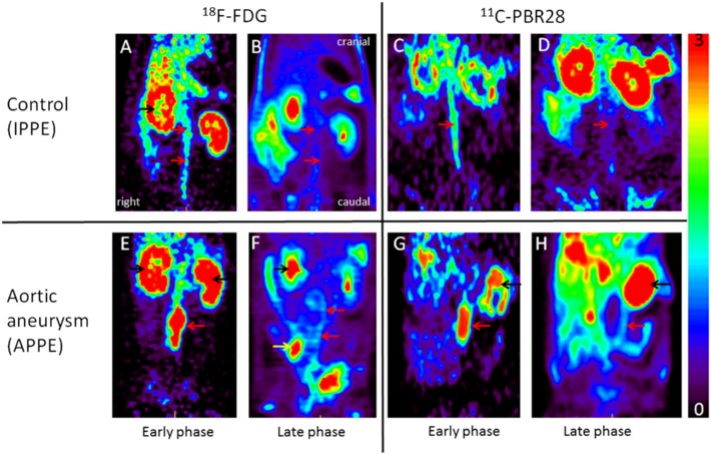
^**18**^ **F-FDG and** ^**11**^ **C-PBR28 microPET.** Representative coronal microPET images of an IPPE control animal **(A-D)** and an APPE abdominal aortic aneurysm (AAA) animal **(E-H)**. Early **(A, E)** and late** (B, F) **phase ^18^ F-FDG scans, with respective early **(C, G)** and late **(D, H)** phase ^11^C-PBR images are shown. Sequential imaging allows for the identification of the aortic wall (red arrows), kidneys (black arrow), and ureteral activity (white arrow).

Figure 2 displays representative ^18^ F-FDG and ^11^C-PBR28 microPET images. Image analysis was complicated, as aortic wall uptake was generally lower than the radioactivity in bowel in both ^18^ F-FDG and ^11^C-PBR28 scans. However, the blood pool activity seen on early phase imaging (0 to 90 s. p.i.) revealed the location of the abdominal aorta and AAAs. On late phase images, AAAs were always visually identifiable on both ^11^C-PBR28 and ^18^ F-FDG microPET data. However, a clear trend favoring one of the two tracers for visualization of AAAs was not observed.

While ^11^C-PBR28 accumulated rapidly in the renal cortex, the renal excretion of radioactivity into the urine, as seen with ^18^ F-FDG, was not observed with ^11^C-PBR28. Dynamic PET imaging, however, improved identification of extra-aortal structures, as radioactivity associated with bowel content and urine was variable over time.

**Figure 3:**
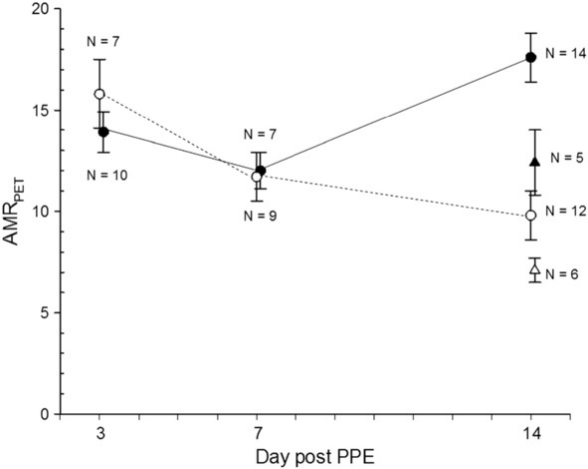
**Time course of** ^**18**^ **F-FDG and** ^**11**^ **C-PBR28 AMR**_**PET**_ **. **The ^18^ F-FDG AMR_PET_ (mean ± SEM) of APPE (black circles, black lines) of AAA animals at day 14 was significantly greater than that at day 3 (p = 0.03) and day 7 (p = 0.002); whereas the ^18^ F-FDG AMR_PET_ of IPPE control animals (white circles, dashed lines) decreased over the 14-day period (p = 0.01 day 3 vs. day 14). APPE AAA animals also demonstrated a significantly greater ^18^ F-FDG AMR_PET_ at day 14 compared to IPPE controls (p = 0.0002). At day 14, the ^11^C-PBR28 AMR_PET_ was significantly greater (p = 0.04) in APPE (black triangles) compared to IPPE controls (white triangles).

Due to unavoidable restrictions regarding the availability of radiotracers and scanner time, imaging could not be performed on all animals at every time point. ^18^ F-FDG microPET at 14 days post PPE exposure (Figure 3) revealed a significantly greater APPE AAA AMR_PET_ (mean ± SEM 17.6 ± 1.2, N = 14) compared to IPPE controls (9.8 ± 1.2, N = 12; p = 0.0002). No significant differences in AMR_PET_ were noted at 3 and 7 days post PPE exposure between APPE and IPPE groups. At day 3 post PPE exposure, the mean ^18^ F-FDG AMR_PET_ for APPE AAAs (N = 7) and IPPE controls (N = 10) were 13.9 ± 1.0 and 15.8 ± 1.7 (p = NS), respectively. At day 7, the mean ^18^ F-FDG AMR_PET_ values for APPE AAAs (N = 7) and IPPE controls (N = 9) were 12.0 ± 0.9 and 11.7 ± 1.2 (p = NS), respectively. Interestingly, the average ^18^ F-FDG uptake (between 60 and 90 min. p.i. in nCi/cm^3^) in the aorta of IPPE control animals was 1.9 times higher than muscle background (p < 0.02).

**Figure 4:**
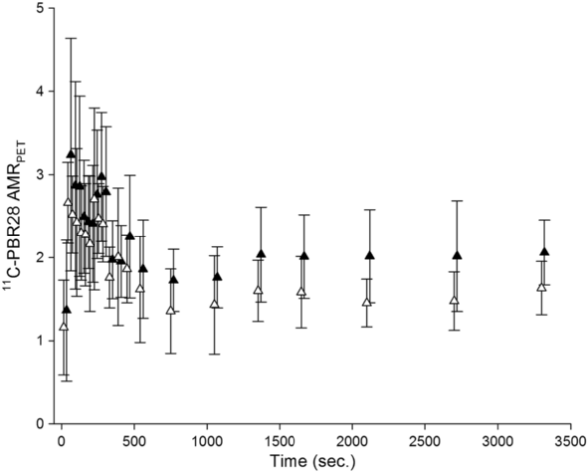
^**11**^ **C-PBR28 time-activity curves.** About 10 min after radiotracer injection, the aorta-to-muscle background ratio (AMR_PET_) for ^11^C-PBR28 was fairly stable and consistently higher in APPE AAAs (black triangles) compared to IPPE control aortas (white triangles). APPE and IPPE data points are separated (in time) for clarity.

^11^C-PBR28 microPET data were not obtained at days 3 and 7 post PPE exposure. Time-activity data obtained from day 14 indicated that the ^11^C-PBR28 AMR_PET_ was fairly stable from 10 to 60 min after tracer injection and that the AMR_PET_ was higher in APPS compared to IPPE controls (Figure 4). In fact when summing data between 10 and 60 min p.i. at day 14, the mean APPE AAA ^11^C-PBR28 AMR_PET_ (12.4 ± 1.6, N = 5) was significantly greater than that of IPPE controls (7.1 ± 1.0, N = 6) (p = 0.04). Also, the average ^11^C-PBR28 uptake (summed between 10 and 60 min. p.i. in nCi/cm^3^) in the aorta of controls was 2.5 times higher than in muscle background (p < 0.001).

**Figure 5:**
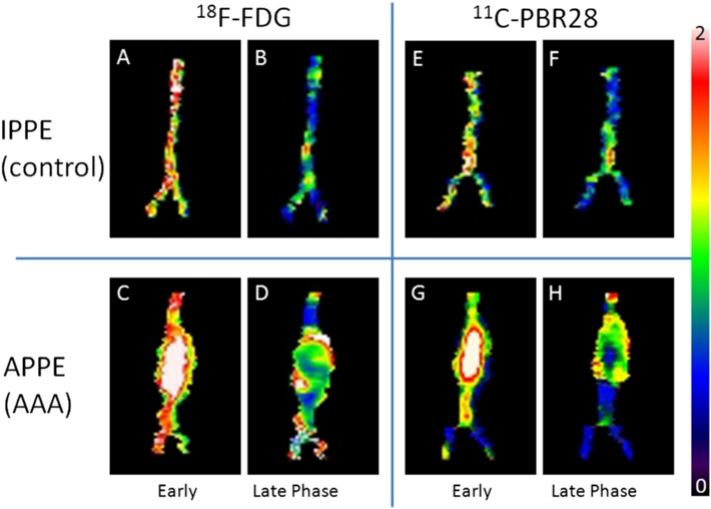
^**18**^ **F-FDG and** ^**11**^ **C-PBR28 maximum intensity projections of the aorta.** Anterior projections of aortal maximum intensity projection (MIP) images are shown from an IPPE control **(A, B, E, F)** in comparison to a APPE AAA animal **(C, D, G, H)**. **(A-D)** display ^18^ F-FDG uptake, while **(E-H)** demonstrate ^11^C-PRB28 uptake at the aortic wall surface (scaled as SUV values; range 0 to 2). The AAA is clearly identified by both radiotracers on early and late phase images. Individual movie files of these data are provided [see Additional files 1, 2, 3, 4, 5, 6, 7 and 8].

Figure 5 shows early and late phase segmentations of the aortic VOIs obtained from representative APPE and IPPE control animals. As seen on anterior projections, the ^18^ F-FDG and ^11^C-PBR28 distribution was homogeneous in controls, but clearly inhomogeneous within the AAA wall, thereby often displaying increased uptake at the anterolateral aspect of the AAA in APPE animals. Additional movie files (scaled to SUV values ranging from 0 to 2) show the above cases from Figure 5 in greater detail (see Additional files 1, 2, 3, 4, 5, 6, 7 and 8).

#### ^18^ F-FDG and ^11^C-PBR28 autoradiography

**Figure 6:**
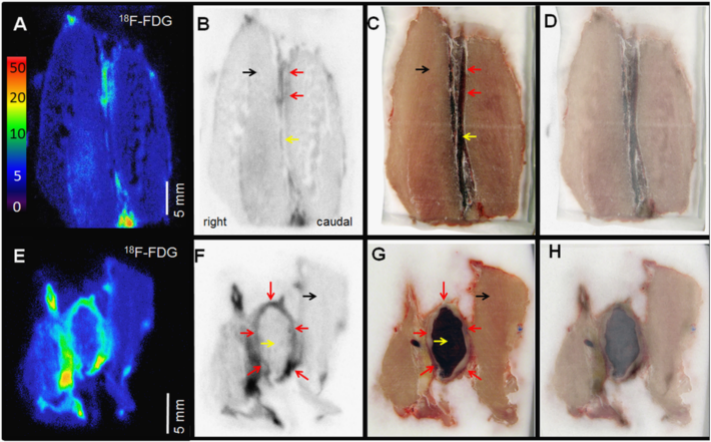
^**18**^ **F-FDG autoradiography.** Representative autoradiography **(A, B, E, F)**, block face photographs **(C, G)** and fusion images** (D, H)** of a representative IPPE control **(A-D)** and an APPE AAA animal **(E-H)**. **(A and E)** represent ^18^ F-FDG uptake (in PSL/mm^2^), while **(B and F)** are gray-scale images displayed with optimal contrast settings for fusion. The ^18^ F-FDG uptake in the aortic wall is increased in the APPE AAA wall (AMR_ARG_ 7.91 ± 1.97) compared to the IPPE control aortic wall (AMR_ARG_ 1.23 ± 0.52). The aortic wall (red arrows), blood pool (yellow arrows), and psoas musculature (black arrows) are identified (scale bar 5 mm).

**Figure 7:**
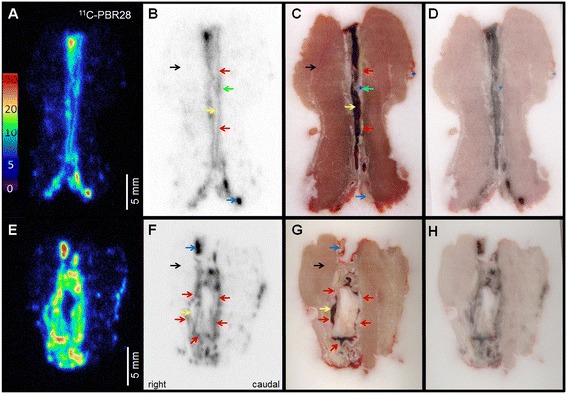
^**11**^ **C-PBR28 autoradiography.** Representative autoradiography **(A, B, E, F)**, block face photographs** (C, G)**, and fusion images **(D, H)** of a representative IPPE control **(A-D)** and an APPE AAA **(E-H)**. Note that the AAA (in E-H) is collapsed causing folds in the aortic wall. ^11^C-PBR28 uptake is increased in the APPE AAA (AMR_ARG_ 28.6 ± 8.0) wall compared to the control aortic wall (AMR_ARG_ 4.7 ± 0.2). The aortic wall (red arrows), blood pool (yellow arrows), psoas musculature (black arrows), suture material (green arrow), and gut activity (blue arrow) are marked.

Analysis of autoradiographic data was not impaired by nearby radioactivity and clearly identified increased ^18^ F-FDG (Figure 6) and ^11^C-PBR28 (Figure 7) uptake in the aortic wall of APPE AAAs compared to IPPE control aortas. As identified on autoradiography, the aortic wall uptake of ^18^ F-FDG and ^11^C-PBR28 was generally nonuniform in APPE AAAs and homogeneous in IPPE controls. In the case shown in Figure 7, mild focal increased ^11^C-PBR28 uptake was noted due to inflammation related to the suture material. The mean AMR_ARG_ for ^18^ F-FDG on day 14 was 9.7 ± 2.3 for APPE AAAs (N = 4) and 2.4 ± 2.0 for IPPE controls (N = 5), respectively (p = 0.02). The mean 14-day AMR_ARG_ for ^11^C-PBR28 was 28.9 ± 4.0 for APPE AAAs (N = 4) and 5.0 ± 0.2 for the IPPE controls (N = 4), respectively (p = 0.01). On average, a 4-fold increase in the aortic wall uptake of ^18^ F-FDG and a 5.8-fold increase in aortic wall uptake of ^11^C-PBR were demonstrated by APPE AAAs compared to IPPE control aortas. While the mean AMR_ARG_ for ^18^ F-FDG (2.4 ± 2.0) and ^11^C-PBR28 (5.0 ± 0.2) in the aortic wall of IPPE control animals was numerically higher than muscle background (set to 1), the difference failed to reach significance for both tracers, which was likely related to the small number of animals investigated.

#### Immunohistochemistry

**Figure 8:**
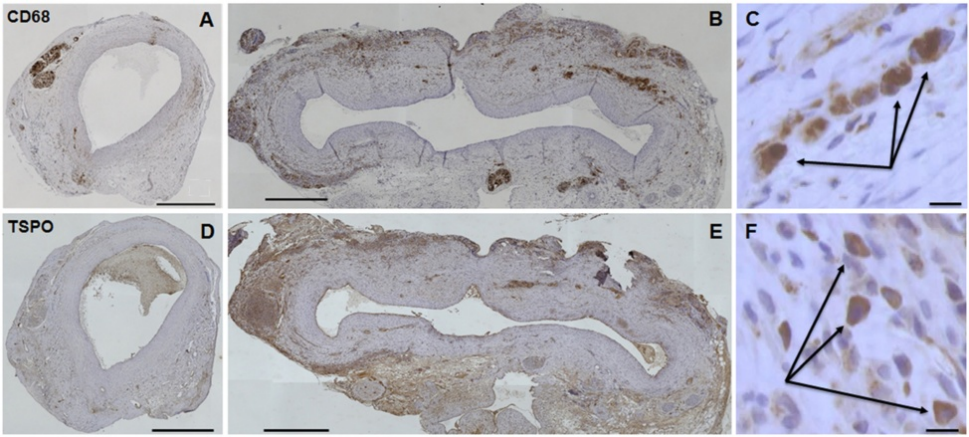
**Immunohistochemistry.** **(A)** IPPE control stained with anti-CD68. **(B)** APPE AAA stained with anti-CD68. **(C)** High-power magnification of macrophages (identified by arrows) stained with anti-CD68. **(D)** IPPE control stained with anti-TSPO. **(E)** APPE AAA stained with anti-TSPO. **(F)** High-power magnification of macrophages (identified by arrows) stained with anti-TSPO. **(A, B, D, E)** × 10 magnification, scale bar 500 μm. **(C, F)** × 63 magnification, scale bar 10 μm.

A significantly greater number of CD68- and TSPO-positive cells were observed in APPE AAA sections compared to IPPE control aortic sections. CD68-positive cells per HPF ratios of 0.95 ± 0.11 and 0.14 ± 0.01 (p = 0.001) were determined in APPE AAAs (N = 6) and IPPE controls (N = 6), respectively. As displayed in Figure 8, TSPO-positive cells per HPF ratios were determined at 0.110 ± 0.018 for APPE AAAs (N = 6) and 0.018 ± 0.003 (p = 0.004) for IPPE controls (N = 4), respectively.

#### Macrophage cell culture

**Figure 9:**
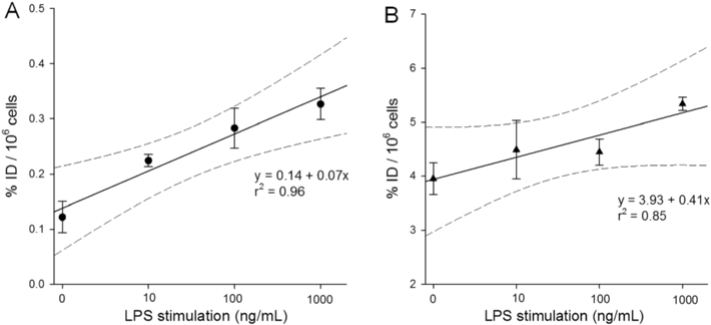
**Macrophage radiotracer uptake.** RAW 264.7 macrophage cell uptake of **(A)** ^18^ F-FDG (dots) and **(B)** ^11^C-PBR28 (triangles). Both radiotracers demonstrate a dose-dependent increase of tracer uptake in response to LPS stimulation (logarithmic scale). Regression analysis results (solid line with dashed 95% confidence intervals) are provided.

Figure 9 displays decay-corrected ^11^C-PBR28 and ^18^ F-FDG RAW 264.7 macrophage cell uptake (expressed as percentage of injected dose/million cells) in response to LPS stimulation. The ^11^C-PBR28 uptake increased in a dose-dependent fashion from nonstimulated macrophages (3.95 ± 0.51) to 5.33 ± 0.38 at 1,000 ng/mL LPS. The ^18^ F-FDG uptake also increased from 0.12 ± 0.04 at 0 ng/mL LPS to 0.33 ± 0.09 at 1,000 ng/mL LPS. Using the same initial mass tracer concentrations (approximately 10 nmol/mL), the macrophage cell uptake of ^11^C-PBR28 was substantially (between 16 and 32 times) higher than that of ^18^ F-FDG at any stimulation level (p < 0.001).

#### Quantitative real-time polymerase chain reaction

At day 14 post PPE exposure, the mean TSPO gene expression was significantly greater for APPE AAAs (N = 14) compared to IPPE controls (N = 4) at 4.6 ± 0.6 × 10^−5^ and 1.3 ± 0.1 × 10^−5^ (p = 0.0002), respectively.

## Discussion

Comparable aortic diameter increases during IPPE and APPE exposure demonstrate the consistency and reproducibility of the model. The greater degree of aortic diameter increase associated with the APPE group, immediately following dilation, compared with the IPPE group demonstrates the subsequent effect of the 30-min exposure to APPE. The ultimate aortic diameter increases at day 14 post APPE exposure are consistent with previous results for this model [7,13]. We interpret the decrease in aortic diameter following IPPE exposure in the control group over the 14-day period as a consequence of the reparative process that the rat aorta undergoes after the mechanical disruption caused by sham treatment.

In the selected well-established animal model [13,20,21], it has been demonstrated that a wider variety of inflammatory cells including neutrophils, T cells, mast cells, and monocytes infiltrate the AAA wall during the acute and transitional phases of AAA development that occur over the first 3 and 7 days, respectively. Of these inflammatory cells, macrophages and T cells maintain a greater presence during the chronic inflammatory phase out to and beyond 14 days post APPE exposure [22]. By performing ^18^ F-FDG and ^11^C-PBR28 microPET imaging and respective autoradiography, with supportive IHC with APPE AAA and IPPE controls, we tested whether noninvasive microPET imaging would allow the evaluation of the inflammatory response to PPE exposure.

The assessment of rodent AAA development by small animal microPET is limited due to the small size of the rat aorta and the potential presence of confounding nearby radioactivity in bowel loops or ureters. We addressed these limitations in part by performing dynamic microPET and by injection of the radiotracers via the jugular vein catheter. As a result, a clear bolus of activity could be seen in the aorta, without interference from activity in the inferior vena cava, providing the necessary anatomical location of the aortic lumen for VOI definition. Using this technique, we identified elevated ^18^ F-FDG uptake in the aortic wall 14 days after APPE exposure compared to controls, while no significant differences were noted at 3 and 7 days. These results were further validated by autoradiography, performed 14 days post PPE exposure, demonstrating significantly greater ^18^ F-FDG uptake in the aortic wall of APPE AAA walls compared to IPPE controls. The decreasing ^18^ F-FDG uptake over the course of time post IPPE exposure correlates with the reparative process represented by continued decreases in aortic diameter in controls. By contrast, 14 days post APPE exposure, the increased ^18^ F-FDG uptake post APPE exposure correlated with the increasing aortic diameter, which is caused by an ongoing inflammatory response. Our inability to differentiate ^18^ F-FDG uptake by IPPE controls and APPE AAAs at 3 and 7 days post PPE exposure likely reflects a combination of the inflammatory processes associated with the operative procedure and transient mechanical aortic disruption that occurs within both groups.

Given that the ^18^ F-FDG uptake in our model has not previously been assessed, comparisons with the literature are limited. Ogawa et al. observed greater aortic uptake on ^18^ F-FDG PET imaging and determined greater ^18^ F-FDG differential uptake ratios for the thoracic and abdominal aortas of Watanabe heritable hyperlipidemic rabbits that developed intimal thickening and plaque formation [23]. Furthermore, increased ^18^ F-FDG uptake was correlated with increased macrophage populations in the atherosclerotic plaques on IHC [23] and predominant T lymphocytes in human AAA undergoing scheduled surgical repair [24]. Recently, Courtois et al. identified increased ^18^ F-FDG uptake associated with active inflammation characterized by infiltrates of proliferating leukocytes in the adventitia of AAAs [25]. We observed increased macrophage populations by CD68 IHC and increased ^18^ F-FDG uptake ratios by autoradiography in AAA walls compared to control aortic walls, however, in separate groups. As measured by microPET, the ^18^ F-FDG uptake in the aorta of sham-treated animals was significantly higher compared to muscle background, which was likely related to the inflammation induced by the surgical trauma.

TSPO binding by PET radioligands has been well investigated with regard to central nervous system inflammation [26-28]. In addition, we have recently shown the utility of ^11^C-PBR28 to visualize extracranial inflammation in acute and chronic animal models based on carrageenan and T cell-mediated adjuvant arthritis, respectively [15]. However, little is known about the utility of TSPO radiotracers to demonstrate aortic wall inflammation. Here, we demonstrate that 14 days after APPE exposure, significantly increased ^11^C-PBR28 uptake is noted in the AAA wall on microPET and autoradiography compared to controls. In addition, we identified significantly increased TSPO expression in APPE AAA walls compared to IPPE control aortas using qRT-PCR.

While TSPO and CD68 IHC as well as ^18^ F-FDG and ^11^C-PBR28 autoradiography were performed in this study, a direct spatial comparison between both was not available due to technical incompatibilities (lack of IHC antibodies suitable for frozen samples). However, spatial co-localization of the TSPO radioligand ^3^H-PK11195 and TSPO IHC has previously been verified by Gaemperli et al. [12]. Also, we previously identified a correlation of ^11^C-PBR28 uptake with CD68 staining scores in carrageenan-induced acute inflammation [15]. These observations provide further supportive evidence that the increased ^11^C-PBR28 uptake in APPE AAAs was indeed related to an increase in TSPO protein expression.

Sarda-Mantel et al. recently evaluated ^18^ F-FDG and the TSPO radioligand ^18^ F-DPA714 to assess AAA wall inflammation utilizing an aortic xenograft model involving orthotopic implantation of decellularized guinea pig abdominal aorta in rats [29]. While the time course for AAA development and the respective inflammatory response associated with this aortic xenograft model are quite different from those associated with the PPE model, their results also indicate a potential role of ^18^ F-FDG and ^18^ F-DPA714 in the assessment of AAA wall inflammation.

In our study, we observed a greater mean AAA ^18^ F-FDG uptake (expressed as AMR_PET_) compared to ^11^C-PBR28 uptake (17.6 ± 1.2 and 12.4 ± 1.6) at day 14 post APPE exposure, however, with greater mean AAA ^11^C-PBR28 uptake (expressed as AMR_ARG_) on autoradiography (28.9 ± 4.0 and 9.7 ± 2.3, respectively) compared to ^18^ F-FDG. It should be noted that a direct comparison of ^11^C-PBR28 and ^18^ F-FDG is difficult due to important technical differences (mainly related to differences in voxel resolution) between microPET and autoradiography and profound differences in the AMR analytic technique between these two modalities (forced by the different physical half-life of ^11^C and ^18^ F). Given the rapid decay of ^11^C-PBR28, whole-mount autoradiography was limited to a subset of coronal slices cut through the center of the AAA; whereas the calculation of AMRs obtained from PET is based on VOIs including the entire aortic wall. In addition, while the longer physical half-life of ^18^ F allowed for similar timing of ^18^ F-FDG PET and autoradiography (at 60 min post injection), the short half-life of ^11^C-PBR28 required sectioning to start much earlier. Since we previously identified rapid kinetics of ^11^C-PBR28 in two other inflammation models [15] and since we saw a significant difference in the ^11^C-PBR28 AMR_PET_ as early as 10 min p.i., a 10-min circulation time was selected for ^11^C-PBR28 autoradiography. Other researchers had also opted for early time point autoradiography (at 20 min. p.i.) when investigating atherosclerotic plaques with ^11^C-PK11195 [11]. Thus, AMRs obtained from PET (AMR_PET_) and autoradiography (AMR_ARG_) were timed differently and not expected to necessarily display similar results. Nevertheless, both measurements (PET and autoradiography) identified increased ^18^ F-FDG and ^11^C-PBR28 uptake in APPE AAAs compared to IPPE controls. The uptake of both tracers was nonuniform within AAAs favoring the anterolateral aspects of aneurysms. It remains to be seen whether this observation is specific to the PPE model. Similar to ^18^ F-FDG, we observed increased ^11^C-PBR28 uptake (AMR_PET_) in IPPE control aortas compared to muscle background. While elevated TSPO radiotracer uptake in healthy mouse arteries has been observed [11], we caution that the elevated ^11^C-PBR28 uptake in IPPE control aortic walls may primarily have been the result of the unavoidable surgical trauma.

TSPO ligands such as ^11^C-PBR28 may provide more specific (or relevant) information about inflammatory cell infiltration into the AAA wall than ^18^ F-FDG. In humans, asymptomatic noninflammatory AAA selected for surgical repair due to size criteria lack significantly increased ^18^ F-FDG uptake on PET while at the same time, focal ^18^ F-FDG uptake is identified on autoradiography co-localizing to focal accumulations of CD45-, CD3-, and CD20-positive leukocytes on IHC with very little contribution of macrophages [24]. However, macrophages play an important role in the AAA wall prior to rupture [30]. Matrix metalloproteinases produced by macrophages (and B cells) degrading collagen and elastin in the abdominal wall have been linked with the continued enlargement of AAAs [3,31]. As mentioned above, TSPO radiotracers such as ^11^C-PBR28 are known for preferential accumulation in macrophage-like glial cells [26,28]. As shown in this study as well as on prior occasion [15], ^11^C-PBR28 also displays elevated uptake in activated macrophages. Given that the macrophage cell uptake of ^11^C-PBR28 was at least one order of magnitude greater than that of ^18^ F-FDG (measured at the same initial mass tracer concentration), one could speculate that the presence (or level) of ^11^C-PBR28 uptake in human AAAs may be of greater clinical relevance than that of ^18^ F-FDG due to its more specific link to macrophage function. This elevated macrophage cell uptake of ^11^C-PBR28, particularly with activation, also raises the possibility that partial volume-effect-related limitations of ^18^ F-FDG in the assessment of human AAAs [32] could potentially be overcome.

Due to different binding mechanisms, the binding affinity of various TSPO radioligands may vary significantly. In humans, it now has been clearly demonstrated that the TSPO protein exists as a monomer as well as part of a multimeric complex consisting of multiple TSPO monomers. Furthermore, human TSPO polymorphism significantly influences ^11^C-PBR28 binding potential [33,34]. To assess binding potential in humans, a simple blood test now evaluates the binding affinity of platelets for ^11^C-PBR28 [35]. While the existence of such a TSPO polymorphism has only been investigated in humans, its existence cannot be excluded in rats and may have inadvertently interfered with our results. However, despite human TSPO polymorphism, the potential for ^11^C-PBR28 PET for assessments of human AAA development, atherosclerosis, and vasculitis appears to be significant [36]. In addition, certain TSPO receptor ligands may exert an anti-inflammatory effect via steroid synthesis that could downregulate pro-inflammatory interleukin secretion. Torres et al. demonstrated decreased acute extremity and pulmonary inflammation in two murine models with administration of PK11195 and Ro5-4864, with specific reductions in IL-6 and IL-13 [37]. Therefore, TSPO radioligands could become useful not only as PET imaging agents to aid in diagnosis but also to monitor anti-inflammatory treatments targeting the TSPO protein.

## Conclusions

For the first time, we have demonstrated the utility of ^18^ F-FDG and ^11^C-PBR28 to study inflammation associated with the PPE model of AAA development in rats, thereby verifying similar ^18^ F-FDG imaging characteristics in this model compared to humans. In conjunction with increased TSPO gene expression and protein expression in AAA, this data supports the role of ^18^ F-FDG and TSPO radioligands for the assessment of aortic wall inflammation during the development of aortic aneurysms in patients. Based on macrophage uptake and autoradiographic findings, as well as the natural history of AAA rupture, we see potential advantages for TSPO radiotracers over ^18^ F-FDG and suggest a clinical trial to assess the value of ^11^C-PBR28 for the prediction of AAA rupture.

## Additional files

Additional file 1:
**Movie file of** ^**18**^ **F-FDG maximum intensity projections of the aorta at early phase (0 to 90 s) of an IPPE control animal.**
Additional file 2:
**Movie file of** ^**18**^ **F-FDG maximum intensity projections of the aorta at late phase (60 to 90 min) of an IPPE control animal.**
Additional file 3:
**Movie file of** ^**11**^ **C-PBR28 maximum intensity projections of the aorta at early phase (0 to 90 s) of an IPPE control animal.**
Additional file 4:
**Movie file of** ^**11**^ **C-PBR28 maximum intensity projections of the aorta at late phase (60 to 90 min) of an IPPE control animal.**
Additional file 5:
**Movie file of** ^**18**^ **F-FDG maximum intensity projections of the aorta at early phase (0 to 90 s) of an APPE AAA animal.**
Additional file 6:
**Movie file of** ^**18**^ **F-FDG maximum intensity projections of the aorta at late phase (60 to 90 min) of an APPE AAA animal.**
Additional file 7:
**Movie file of** ^**11**^ **C-PBR28 maximum intensity projections of the aorta at early phase (0 to 90 s) of an APPE AAA animal.**
Additional file 8:
**Movie file of** ^**11**^ **C-PBR28 maximum intensity projections of the aorta at late phase (60 to 90 min) of an APPE AAA animal.**

## Abbreviations

^11^C-PBR28: N-acetyl-N-(2-[^11^C]methoxybenzyl)-2-phenoxy-5-pyridinamine
^18^ F-FDG: 2-deoxy-2-[^18^ F]fluoro-d-glucose
AAA: abdominal aortic aneurysm
APPE: active porcine pancreatic elastase
AMR: aortic wall-to-muscle ratio
AMR_ARG_: aortic wall-to-muscle ratio (on autoradiography)
AMR_PET_: aorta-to-muscle ratio (on PET)
HPF: high-power field
IHC: immunohistochemistry
IPPE: heat-inactivated porcine pancreatic elastase
MicroPET: micro-positron emission tomography
MIP: maximum intensity projection
OSEM-MAP: ordered subset expectation maximization - maximum a posteriori
PPE: porcine pancreatic elastase
RAAA: ruptured AAA
TSPO: translocator protein (formerly peripheral benzodiazepine receptor)
US: ultrasound
VOI: volume of interest
